# Reproducibility of cardiac volumetric parameters derived from fully automatically prescribed image planes: a direct comparison to manual planning at 1.5-T and 3-T MRI

**DOI:** 10.1007/s00330-026-12388-9

**Published:** 2026-02-21

**Authors:** Karolin K. Deyerberg, Felix G. Meinel, Lena-Maria Watzke, Ann-Christin Klemenz, Mathias Manzke, Margarita Gorodezky, Gaspar Delso, Antonia Dalmer, Roberto Lorbeer, Danagul Zhexenova, Marc-André Weber, Benjamin Böttcher

**Affiliations:** 1 Institute of Diagnostic and Interventional Radiology, Pediatric Radiology and Neuroradiology, Rostock University Medical Center, Rostock, Germany; 2 GE HealthCare, Munich, Germany; 3 GE HealthCare, Barcelona, Spain; 4 Department of Radiology, Ludwig-Maximilian University, Munich, Germany; 5 University Medical Center, Astana, Kazakhstan

**Keywords:** Cardiac, Magnetic resonance imaging, Automated workflow, Artificial intelligence, Reproducibility

## Abstract

**Objectives:**

The prescription of cardiac MRI (CMR) image planes is essential for comparable volumetric assessment, but manual planning is time-consuming and error-prone. This prospective single-center study evaluated automated planning and its impact on the reproducibility of volumetric parameters derived from CMR.

**Materials and methods:**

Fifty-two healthy volunteers (26 males, median age 44.5 years) were divided into a 1.5 T sub-cohort (*n* = 32, both scans at 1.5 T, interval 2–5 weeks) and a 3 T sub-cohort (*n* = 20, 1st scan 1.5 T, 2nd scan 3 T, interval 1–2 h). All scans were performed using automated and manual planning with identical protocols, acquiring standard cardiac planes. Subjective quality of plane position was rated blinded by two radiologists. Volumetric analysis was performed fully automatically without corrections on SAX, retrieving right ventricular (RV) and left ventricular (LV) parameters. Wilcoxon matched-pairs signed rank test, intraclass correlation coefficient (ICC), and Bland–Altman analysis were used for statistical assessment.

**Results:**

Subjective quality of image planes showed high consistency with good to excellent ratings in both sub-cohorts. Reproducibility of volumetric parameters was good to excellent (all ICC > 0.77) except for LVEF (1.5 T sub-cohort: LVEF manual: 0.323; automated: 0.213; 3 T sub-cohort: LVEF manual: 0.597; automated: 0.742). Overall, reproducibility was better in the 3 T sub-cohort, mainly due to different scan intervals. ICCs were slightly higher compared to manual planning across both sub-cohorts. These trends were also observed in the Bland–Altman analysis.

**Conclusion:**

Fully automated plane positioning for CMR provides high-quality image planes, ensuring high reproducibility of cardiac volumetric parameters across both established field strengths.

**Key Points:**

***Question*** *The prescription of CMR image planes is essential for a comparable volumetric cardiac analysis, but manual planning is time-consuming and error-prone*.

***Findings*** *Automated plane prescription for CMR provides high-quality image planes, ensuring high reliability and reproducibility of cardiac volumetric parameters across both established field strengths*.

***Clinical relevance*** *Automated plane prescription for CMR reliably provides high-quality image planes, ensuring comparable cardiac volumetric parameters. This technology can simplify the acquisition and promises to reduce variability between follow-up scans, as well as to enhance the availability for patients*.

**Graphical Abstract:**

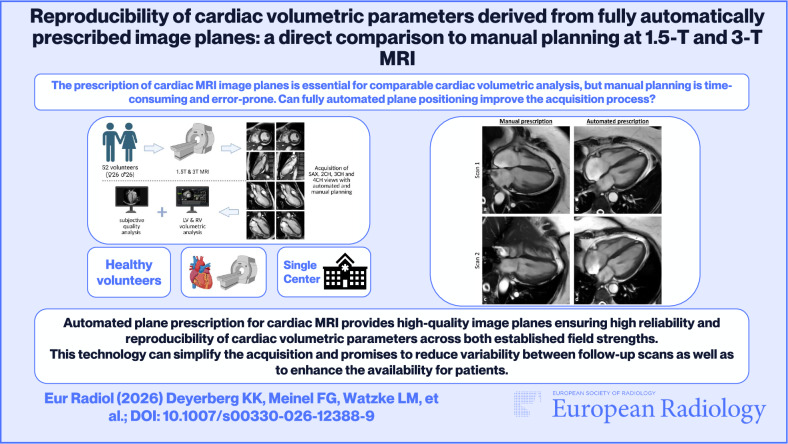

## Introduction

Cardiac magnetic resonance imaging (CMR) is an essential part of the diagnostic process for a wide range of cardiovascular diseases [1]. It is the current gold standard for the quantitative assessment of cardiac volumes, myocardial function, and tissue characterization, and is therefore recommended in various international and interdisciplinary guidelines [2–5]. For volumetric analysis, CMR showed improved reproducibility and validity compared to echocardiography [6–8]. However, a certain marginal variability between repeated measurements remains, mainly attributable to technical and physiological factors [9, 10].

The reproducibility of CMR data is of central importance in follow-up scans of various cardiac diseases, such as myocarditis [5], Tako Tsubo [11, 12], or cardiomyopathies [6, 7]. It is vital to quantify pathological changes precisely and consistently in order to guide therapeutic decisions and assess disease progression [13]. Previous strategies to improve reproducibility have focused on technician training guidelines [14], technical regularization [15], including standardization of sequence protocols [16] and post-processing [17, 18]. Further, the integration of artificial intelligence (AI)-based applications into the various components of the CMR workflow is leading to an increased level of automation [19].

At the point of CMR image acquisition, prescription of cardiac image planes significantly determines the quality of the acquired image series and volumetric assessment. State-of-the-art plane positioning for CMR is performed manually, and early investigations showed a high level of reproducibility for manual planning when performed by experienced technicians [20]. However, the need for specially trained personnel [14], the temporal demands, and errors during manual planning remain a significant limitation of CMR, resulting in limited accessibility and restricted availability at specialized centers.

The automation of CMR plane prescription utilizing AI-based systems promises to increase standardization of CMR image acquisition [21**–**24]. This has the potential to reduce variability between follow-up scans and simplify the planning procedure, which can increase the availability of CMR [24]. However, automated plane prescription needs to maintain the high level of reproducibility of the established manual planning, and literature on the reproducibility of cardiac volumetric parameters derived from automated prescribed image planes is scarce [21, 22].

The purpose of this study was to compare the reproducibility of cardiac volumetric assessment derived from fully automatically prescribed CMR images against state-of-the-art manual planning in a test-retest setting, incorporating both established field strengths.

## Material and methods

### Ethical approval and participant selection

This prospective single-center cohort study was granted ethical approval by the institutional review board of the University Medical Center Rostock in line with the Declaration of Helsinki.

The study cohort comprised healthy adult subjects, including both genders, with a minimum age of 18 years and the capability of giving written informed consent. Exclusion criteria were defined as follows: presence of functional or structural heart diseases that directly or indirectly affect the heart, chronic lung or systemic disease (e.g., COPD, rheumatic diseases, etc.), pregnancy, or general contraindications for a magnetic resonance imaging (MRI) examination, such as non-MRI-capable implants.

The volunteers were selected based on equal gender distribution within the following predefined age groups: ≤ 34 years, 35–44 years, 45–54 years, and ≥ 55 years. Demographic data were documented prior to the first CMR scan. The heart rate was recorded during the examinations.

### Study design and MRI protocol

**Fig. 1:**
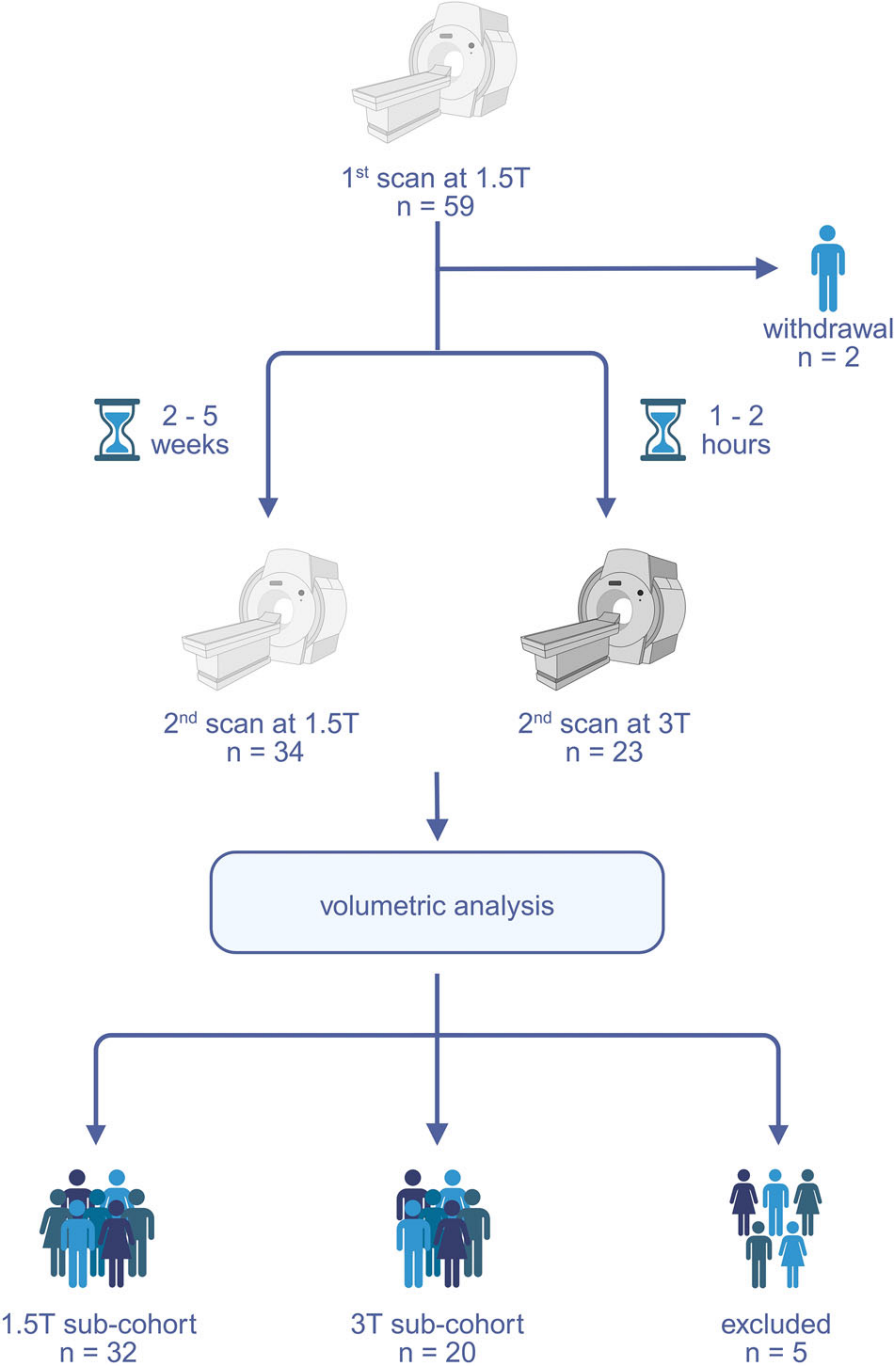
Study design and cohort. Overview of the study design and both sub-groups. The 1.5 T sub-cohort received Scans 1 and 2 at 1.5-T field strength with an inter-scan interval ranging from two to five weeks. The 3 T sub-cohort received Scan 1 at 1.5-T and Scan 2 at 3-T field strength with an inter-scan interval ranging from 1 to 2 h. Two volunteers withdrew from participation before the second CMR scan. Five other volunteers were excluded due to incomplete coverage of the LV in the manual prescribed SAX image stacks, which resulted lack of a reference standard for volumetric results. The final cohort included 52 volunteers. Figure created with BioRender (https://app.biorender.com/)

The study design incorporates two identical CMR examinations. All participants underwent the first scan (hereinafter referred to as Scan 1) on a 1.5-T scanner (SIGNA™ Artist, GE HealthCare). For the second scan (hereinafter referred to as Scan 2) two sub-cohorts were established, as visualized in Fig. 1. The first subgroup (1.5 T sub-cohort) was established to assess the intra-field strength reproducibility and received the second CMR scan (Scan 2) on the same 1.5-T scanner used for Scan 1, with a time interval of two to five weeks. The scan interval was established with the objective of representing a clinically realistic interval. The second subgroup (3 T sub-cohort) aimed to assess the inter-field strength reproducibility, and Scan 2 was performed on a 3-T scanner (SIGNA™ Premier, GE HealthCare) directly after Scan 1 with a time interval of 1–2 h. The volunteers of the 3 T sub-cohort were not allowed to eat or drink between the two CMR scans to avoid any side effects on the cardiac function.

The MRI protocol consisted of accelerated cine sequences (Sonic DL^TM^, GE HealthCare) with a three-heartbeat acquisition (3RR) and breath-holds [25], acquiring standard cardiac imaging planes including a left ventricular (LV) short-axis image stack (SAX) and single image planes in 2-chamber (2CH), 3-chamber (3CH), and 4-chamber view (4CH). Fully automated plane positioning was performed first, utilizing a prototype tool (AIRxHeart Prototype, GE HealthCare). Subsequently, the same cardiac image planes were acquired with manual prescription according to established imaging guidelines [16]. MR-protocol parameters for 1.5-T and 3-T field strength are summarized in Table 1.

**Table 1:** MRI protocol parameters at 1.5 T and 3  T

MRI protocol parameters	1.5 T	3 T
Field of view	340 mm^2^	340 mm²
In-plane resolution	1.7 × 1.5 mm^2^	1.7 × 1.5 mm²
Image pixel matrix	200 × 224	200 × 224
Slice thickness	8 mm	8 mm
Spacing	10 mm	10 mm
Frames per cardiac cycle	30	30
TR	3.2 ms	3.1 ms
TE	1.2 ms	1.1 ms
Flip angle	55°	55°
Acceleration	6	6

MRI protocol parameters used for the accelerated cine sequences (Sonic DL^TM^, GE HealthCare) with three heartbeat acquisitions and breath-holds at 1.5-T and 3-T field strength

### Automated AI-based plane prescription

The prototype tool for automated plane prescription (AIRxHeart Prototype, GE HealthCare) is based on a deep learning algorithm that can determine anatomical landmarks, such as the LV apex or the mitral valve. The algorithm first detects the heart position using the 3-plane localizer sequence. Anatomical landmarks, such as the mitral valve and LV apex, are then extracted from subsequent series using deep learning models that generate heatmaps indicating each landmark’s location. Although landmark detection is performed on individual image planes, this information is propagated and iteratively refined throughout the planning process. At each stage, the updated landmark positions are used to prescribe the geometry for the next acquisition. The user retains full control and can review or adjust the prescribed plane position and orientation at any time. The prototype version used in this study is capable of fully automated prescription of transverse image stacks, as well as pseudo-2CH, pseudo-4CH, SAX, 2CH, 3CH, and 4CH views.

### Subjective image assessment

**Fig. 2:**
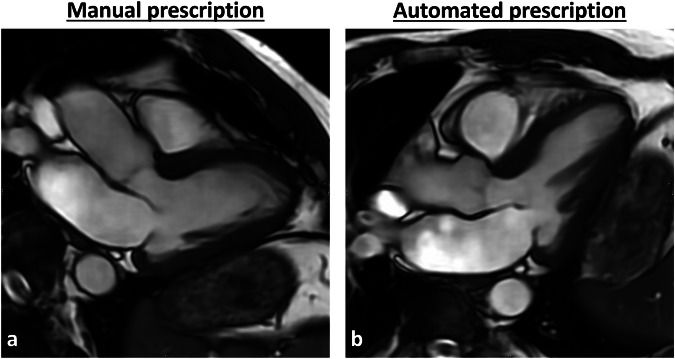
Example of subjective quality rating criteria. Accelerated cine sequences in 3CH view of a healthy 56-year-old male acquired at 1.5 T. **a** Manually prescribed image plane with excellent quality of plane orientation and a subjective rating of 5/5. **b** Automatically prescribed image plane with a good subjective quality rating of 4/5. The aortic root and proximal ascending aorta are not perfectly visualized, but with no diagnostic impairments. This example demonstrates that, when evaluating the subjective plane orientation quality, both readers give a 5/5 rating only if the image plane has been prescribed perfectly. Therefore, the difference between a good (4/5) rating and a perfect (5/5) rating is not relevant for the diagnostic quality. 3CH, 3-chamber-view

The subjective quality of plane orientation was evaluated by two independent radiologists, each with a minimum of five years of experience in CMR, using an in-house browser-based tool in the same way described in a previous publication [26]. Examples of excellent and good quality image planes are visualized in Fig. 2. The results of the subjective quality assessment were analyzed for each plane separately and overall planes for both readers combined.

### Volumetric analysis

Volumetric analysis of the LV and RV was performed fully automatically on SAX series using an established post-processing software (Cvi42, version 5.16, Circle Cardiovascular Imaging Inc.). The automatically generated contours were visually checked for plausibility, but no manual corrections were made. The papillary muscles of the LV were assigned to the blood pool, and the smooth segmentation preset was used. For quantitative assessment, the following parameters were determined for the LV and RV: end-diastolic volume (EDV), end-systolic volume (ESV), stroke volume (SV), and ejection fraction (EF). Additionally, the diastolic mass of the LV was documented.

### Statistical analysis

The statistical analysis was conducted with GraphPad Prism (version 10.3.1, GraphPad Software).

Verification of normal distribution using the Shapiro-Wilk test showed that body weight, height, and heart rate were normally distributed, while the majority of LV and RV volumetric parameters were not. Therefore, the non-parametric Wilcoxon Matched Pairs Signed Rank Test was used under a significance level of *p* < 0.05. Bland–Altman analysis was performed for the LV and RV volumetric parameters. The intraclass correlation coefficient (ICC) was used to assess the test-retest reproducibility of volumetric results derived from each planning approach. ICC values < 0.5 are considered poor, between 0.5 and 0.75 fair, between 0.75 and 0.9 good, and > 0.9 excellent [27, 28].

## Results

### Study population

A total of 57 volunteers completed both CMR scans (1.5 T sub-cohort (Scan 1 and Scan 2 at 1.5 T): *n* = 34; 3 T sub-cohort (Scan 1 at 1.5 T, Scan 2 at 3 T): *n* = 23). The datasets of five participants were excluded because the LV basis was not covered sufficiently in the manual-prescribed SAX image stacks, and therefore, there was no reference standard for volumetric results. The final overall study population consisted of 52 healthy volunteers (26 males, 26 females) with a 1.5 T sub-cohort of *n* = 32 (17 males, 15 females) and a 3 T sub-cohort of *n* = 20 (9 males, 11 females). The overall median age was 44.5 years (range: 23–71), mean weight 77.7 kg (standard deviation: ±12.5 kg), and mean height 1.76 m (standard deviation: ±0.1 m). The mean heart rate was 68.5 bpm with a standard deviation of ±11.9 bpm. The demographic characteristics of both sub-cohorts are outlined in Supplemental Table 1.

### Subjective image analysis

The results of the subjective quality assessment of plane orientations are summarized in Table 2. Overall, the combined subjective quality ratings of both readers (R1 + R2) showed good to excellent results and were comparable between manual and fully automated planning, as well as between both sub-cohorts.

**Table 2:** Subjective quality ratings

Combined subjective quality ratings of image plane orientation						
	Manual prescription			Automated prescription		
	Scan 1	Scan 2	*p*-value	Scan 1	Scan 2	*p*-value
1.5 T sub-cohort (Scan 1 = 1.5 T, Scan 2 = 1.5 T)						
SAX	4.8	4.9	*0.343*	4.8	5	*0.003*
2CH	4.4	4.5	*0.345*	4.5	4.6	*0.624*
3CH	4.4	4.7	*0.082*	4.7	4.7	*0.645*
4CH	4.6	4.7	*0.716*	4.8	4.7	*0.585*
Overall	4.5	4.6	*0.028*	4.6	4.7	*0.235*
3 T sub-cohort (Scan 1 = 1.5 T, Scan 2 = 3 T)						
SAX	4.8	4.9	*0.062*	4.8	5	*0.015*
2CH	4.4	4.5	*0.550*	4.2	4.0	*0.327*
3CH	4.7	4.7	*0.656*	4.6	4.5	*0.467*
4CH	4.7	4.5	*0.509*	4.7	4.6	*0.717*
Overall	4.6	4.6	*0.446*	4.5	4.5	*0.487*

Subjective image quality ratings of both readers are displayed in mean average values (scale from 1 “not diagnostic” to 5 “excellent” image quality). Statistical significance values are presented as *p*-values calculated with the Wilcoxon matched pairs signed rank test (level of significance *p* < 0.05)

*SAX* short-axis, *2CH* 2-chamber-view, *3CH* 3-chamber-view, *4CH* 4-chamber-view

**Fig. 3:**
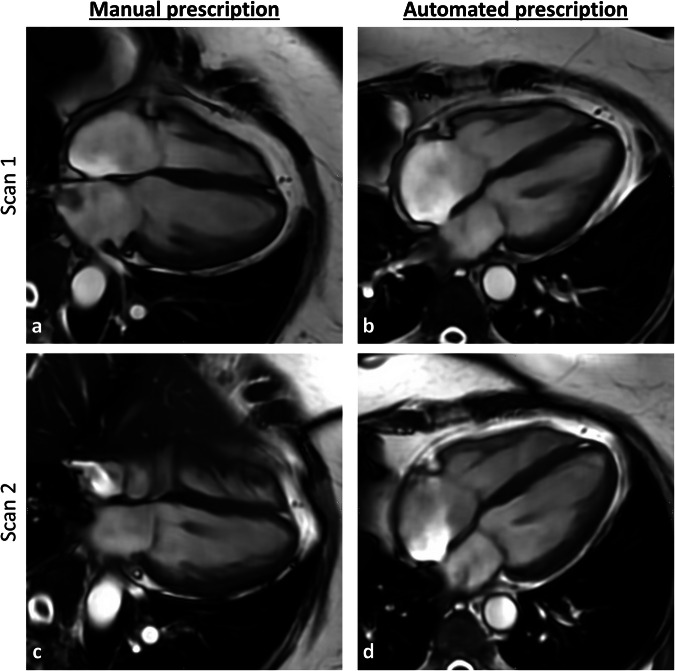
Example of follow-up image planes. Accelerated cine sequences in 4CH view of a healthy 59-year-old female. **a**, **b** The first scan was performed at 1.5 T. Both prescription approaches provided excellent quality (5/5) of the 4CH image plane. **c**, **d** The second scan was performed at 3 T. Manual prescription (**c**) provided a non-diagnostic quality (1/5) of the 4CH initially, requiring a repetition of the cine sequence acquisition or, if not re-acquired, an impaired comparability between both scans. Automated prescription (**d**) demonstrated a high reproducibility in this case. 4CH, four-chamber view

In the 1.5 T sub-cohort, statistically significant differences were only documented for the fully automatically prescribed SAX (Scan 1: 4.8/5; Scan 2: 5/5; *p* = 0.003) and the combined ratings for the manual prescription (Scan 1: 4.5/5; Scan 2: 4.6/5; *p* = 0.028). Similar observations were made in the 3 T sub-cohort with only a single statistically significant difference for the automatically prescribed SAX (Scan 1: 4.8/5; Scan 2: 5/5; *p* = 0.015). However, in absolute values, these ratings vary between 0.1 and 0.2 points and were all located in the good or excellent rating category. The lowest quality ratings were observed for the automatically prescribed 2CH view acquired in Scan 2, with 4/5. The subjective quality ratings from R1 and R2 showed more discrepant results in Scan 2 (R1: 3.4/5; R2: 4.5/5) compared to the automatically prescribed 2CH view in Scan 1 (R1: 4.1/5; R2: 4.3/5). Figure 3 visualizes an example from manual and automated planned 4CH imaging planes.

### Volumetric analysis

The LV and RV volumetric parameters derived from manual and fully automatically prescribed LV SAX image stacks are summarized for the 1.5 T sub-cohort in Table 3 and for the 3 T sub-cohort in Table 4. All datasets successfully passed the visual plausibility assessment of the fully automated generated cardiac contours, meaning that no data need be excluded.

**Table 3:** Volumetric parameters of the 1.5 T sub-cohort

SAX volumetric results 1.5 T sub-cohort								
	Manual prescription				Automated prescription			
	Scan 1	Scan 2	*p*-value	ICC	Scan 1	Scan 2	*p*-value	ICC
Left ventricle (LV)								
LVEDV (ml)	160.5 (114; 245)	157.8 (112; 225.4)	*0.304*	0.962	163 (121; 249)	160.3 (116; 225.8)	*0.239*	0.938
LVESV (ml)	64 (42; 105)	67.5 (39; 119.7)	*0.155*	0.815	66.5 (50; 96)	68 (38; 103.6)	*0.940*	0.766
LVSV (ml)	92.5 (72; 150)	88.2 (70; 147.9)	*0.059*	0.849	96 (70; 152)	92.9 (68; 144.5)	*0.064*	0.894
LVEF (%)	60.5 (46; 69)	57.1 (44; 66.2)	*0.100*	0.323	59.5 (53; 69)	59.4 (43.6; 68.9)	*0.601*	0.213
LV Mass (g)	99.5 (61; 162)	100 (59; 146.2)	*0.089*	0.978	103.5 (68; 159)	98.4 (62; 147.2)	*0.654*	0.984
Right ventricle (RV)								
RVEDV (ml)	171 (113; 289)	165.4 (123; 282.5)	*0.064*	0.969	168.5 (120; 300)	167.6 (112; 265.6)	*0.096*	0.952
RVESV (ml)	76 (40; 142)	81.1 (35; 148.2)	***0.019***	0.936	78 (40; 149)	78.2 (35; 157.9)	*0.178*	0.941
RVSV (ml)	90 (65; 148)	84.9 (59; 137.6)	***< 0.001***	0.848	94.5 (71; 151)	86.8 (60; 141.7)	***0.011***	0.817
RVEF (%)	55 (38; 68)	51.8 (39; 71.4)	***0.001***	0.670	56 (46; 72)	54 (39; 68.2)	***0.032***	0.629

Volumetric parameters of the 1.5 T sub-cohort (Scan 1 and Scan 2 at 1.5 T, *n*** = **32) derived from SAX image stacks. Values are presented in medians with corresponding minimum and maximum values. Statistical significance values are presented as *p*-values calculated with the Wilcoxon matched pairs signed rank test (*p*-values below the level of significance *p*** < **0.05 are shown in bold). In addition, the ICC was specified

*LV* left ventricle, *RV* right ventricle, *EDV* end-diastolic volume, *ESV* end-systolic volume, *SV* stroke volume, *EF* ejection fraction

**Table 4:** Volumetric parameters of the 3 T sub-cohort

SAX volumetric results 3 T sub-cohort								
	Manual prescription				Automated prescription			
	Scan 1	Scan 2	*p*-value	ICC	Scan 1	Scan 2	*p*-value	ICC
LV								
LVEDV (ml)	151.5 (101; 190)	139.7 (95.1; 184.7)	*0.101*	0.972	146 (97; 201)	141.6 (102; 207.7)	*0.576*	0.981
LVESV (ml)	61.5 (36; 96)	57.2 (33.4; 87)	*0.263*	0.915	61.5 (36; 100)	56.8 (37.4; 112.5)	*0.502*	0.957
LVSV (ml)	87.5 (58; 118)	84.4 (61.7; 112.5)	*0.601*	0.893	85.5 (61; 121)	85.9 (64.6; 116.9)	*0.433*	0.918
LVEF (%)	59 (50; 65)	59.2 (50.5; 67)	*0.526*	0.597	59 (47; 65)	58.5 (45.8; 65.7)	*0.502*	0.742
LV Mass (g)	91 (60; 141)	91.8 (62.6; 139)	*0.654*	0.994	91.5 (63; 144)	90.7 (61.9; 141.5)	*0.601*	0.992
RV								
RVEDV (ml)	161 (92; 194)	155.5 (83.5; 225.5)	*0.526*	0.945	160 (90; 211)	154.6 (91.4; 217.4)	*0.576*	0.977
RVESV (ml)	74 (38; 134)	71.3 (36.3; 135.8)	*0.765*	0.979	73.5 (38; 132)	69 (35.2; 128.4)	*0.794*	0.976
RVSV (ml)	80 (53; 105)	80.7 (43.9; 104.9)	*0.455*	0.822	85.5 (53; 116)	84.7 (53.2; 115.9)	*0.126*	0.918
RVEF (%)	54 (31; 63)	53.2 (39.8; 68.3)	*0.576*	0.892	55.5 (38; 65)	53.7 (40.9; 68)	*0.351*	0.905

Volumetric parameters of the 3 T sub-cohort (Scan 1 at 1.5 T, Scan 2 at 3 T, *n*** = **20) derived from SAX image stacks. Values are presented in medians with corresponding minimum and maximum values. Statistical significance values are presented as *p*-values calculated with the Wilcoxon matched pairs signed rank test (level of significance *p* < 0.05). In addition, the ICC was specified

*LV* left ventricle, *RV* right ventricle, *EDV* end-diastolic volume, *ESV* end-systolic volume, *SV* stroke volume, *EF* ejection fraction

In the 1.5 T sub-cohort, following RV parameters reached statistical significance: RVESV (76 vs 81.1 ml; *p* = 0.019), RVSV (90 vs 84.9 ml; *p* < 0.001), and RVEF (55 vs 51.8%; *p* = 0.001) derived from manually prescribed SAX images, and RVSV (94.5 vs 86.8 ml; *p* = 0.011) and RVEF (56 vs 54%; *p* = 0.032) derived from automatically prescribed SAX images. This trend was not observed in the 3 T sub-cohort, where no significant differences were detected neither for the LV nor for the RV volumetric parameters for both planning approaches.

The ICC values showed comparable levels of reproducibility for both manual and automated planning in the 1.5 T sub-cohort without a specific trend favoring one planning approach. There was excellent reproducibility for LVEDV, LV myocardial mass, RVEDV, and RVESV (all ICC > 0.9) and a good reproducibility for LVESV, LVSV, and RVSV (all 0.9 > ICC > 0.75). However, the ICC values for LVEF were < 0.5 for both planning approaches (manual: ICC 0.323; automated: ICC 0.213), indicating poor reproducibility.

In the 3 T sub-cohort, ICC values were consistently higher compared to the 1.5 T sub-cohort, with a similar pattern between manual and automated prescriptions. There was excellent reproducibility for LVEDV, LVESV, LV myocardial mass, RVEDV, and RVESV (all ICC > 0.9) for both planning methods. ICC values of the LVSV, RVSV, and RVEF derived from automatically prescribed SAX images were higher compared to the manual prescription, showing excellent reproducibility. The reproducibility of the LVEF in the 3 T sub-cohort was fair for both planning approaches, with a clear trend favoring the automated prescription (manual: 0.597; automated: 0.742).

### Bland–Altman analysis

**Fig. 4:**
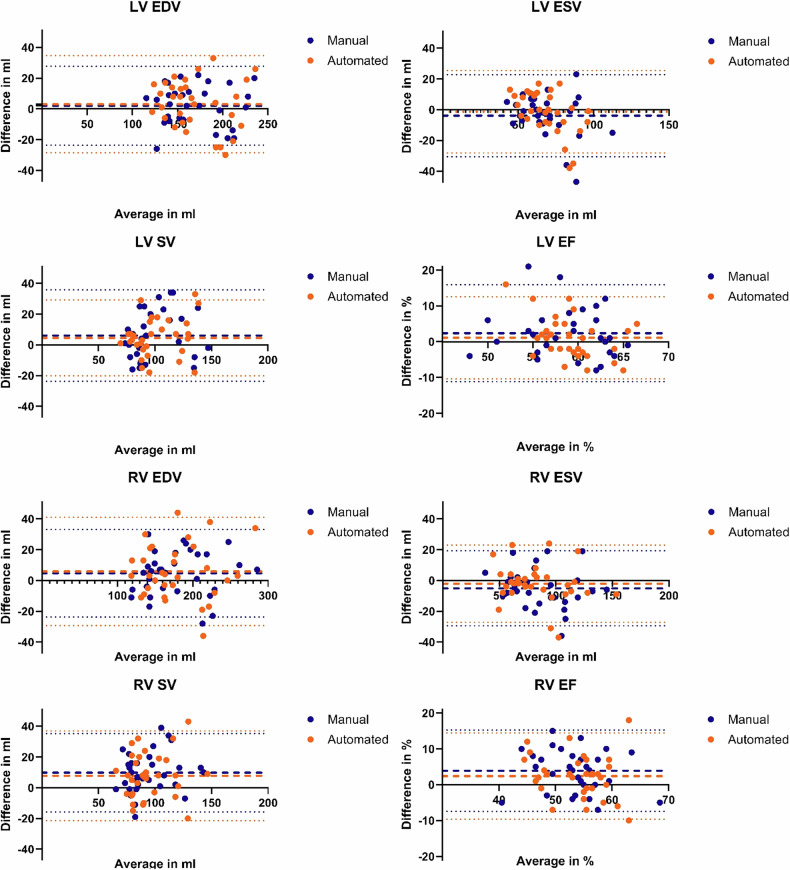
Bland–Altman plots for LV and RV volumetric parameters in the 1.5 T sub-cohort. 1.5 T sub-cohort (Scan 1 and Scan 2 at 1.5 T): Bland–Altman plots for LV and RV volumetric parameters derived from SAX image stacks. The thick dashed line represents the mean bias, the matching colors represent the dotted lines, the 95% limits of agreement. LV, left ventricle; RV, right ventricle; EDV, end-diastolic volume; ESV, end-systolic volume; SV, stroke volume; EF, ejection fraction

**Fig. 5:**
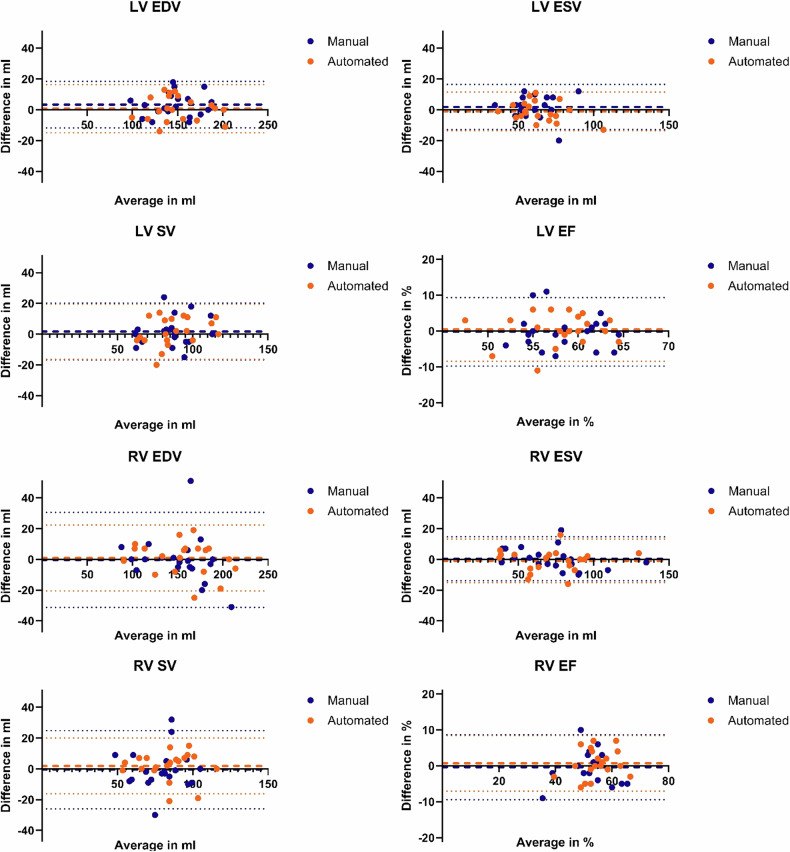
Bland–Altman plots for LV and RV volumetric parameters in the 3 T sub-cohort. 3 T sub-cohort (Scan 1 at 1.5 T, Scan 2 at 3 T): Bland–Altman plots for LV and RV volumetric parameters derived from SAX image stacks. The thick dashed line represents the mean bias, the matching colors represent the dotted lines, and the 95% limits of agreement. LV, left ventricle; RV, right ventricle; EDV, end-diastolic volume; ESV, end-systolic volume; SV, stroke volume; EF, ejection fraction

The results of the Bland–Altman analysis are shown in Table 5. A visual representation of the results can be found in Bland–Altman plots in Fig. 4 for the 1.5 T sub-cohort and in Fig. 5 for the 3 T sub-cohort.

**Table 5:** Bland–Altman analysis of the 1.5 T and 3 T sub-cohort

Bland–Altman analysis of volumetric parameters						
	Manual prescription Scan 1 vs Scan 2			Automated prescription Scan 1 vs Scan 2		
	Bias	SD of bias	95% limits of agreement	Bias	SD of bias	95% limits of agreement
1.5 T sub-cohort						
LV						
LVEDV (ml)	2.0	13.10	-23.6/27.7	3.0	16.12	-28.5/34.6
LVESV (ml)	-3.9	13.63	-30.6/22.8	-1.3	13.6	-28.2/25.4
LVSV (ml)	6.0	15.1	-23.7/35.7	4.5	12.6	-20.1/29.2
LVEF (%)	2.3	6.8	-11.1/15.9	1.0	5.8	-10.3/12.5
RV						
RVEDV (ml)	4.6	14.5	-23.8/33.0	5.8	17.9	-29.3/41.0
RVESV (ml)	-5.1	12.4	-29.4/19.2	-2.1	12.7	-27.1/22.9
RVSV (ml)	9.7	13.0	-15.7/35.3	7.7	14.9	-21.4/37.0
RVEF (%)	3.9	5.7	-7.4/15.2	2.4	6.1	-9.6/14.4
3 T sub-cohort						
LV						
LVEDV (ml)	3.3	7.6	-11.7/18.4	0.7	7.9	-14.9/16.3
LVESV (ml)	1.8	7.5	-12.9/16.5	-1.0	6.4	-13.6/11.5
LVSV (ml)	1.7	9.4	-16.7/20.2	1.6	9.1	-16.3/19.5
LVEF (%)	-0.2	4.8	-9.7/9.3	0.4	4.5	-8.4/9.3
RV						
RVEDV (ml)	-0.3	15.7	-31.2/30.6	0.9	10.9	-20.5/22.3
RVESV (ml)	0.3	7.3	-14.0/14.7	-0.9	7.2	-15.1/13.2
RVSV (ml)	-0.5	12.96	-25.9/24.8	1.9	9.2	-16.2/20.0
RVEF (%)	-0.4	4.6	-9.4/8.6	0.7	3.9	-7.0/8.4

Bland–Altman analysis of volumetric parameters comparing the first and second scan with manual and automated planning for the 1.5 T sub-cohort (Scan 1 and Scan 2 at 1.5 T) and 3 T sub-cohort (Scan 1 at 1.5 T, Scan 2 at 3 T)

*SD* standard deviation, *LV* left ventricle, *RV* right ventricle, *EDV* end-diastolic volume, *ESV* end-systolic volume, *SV* stroke volume, *EF* ejection fraction

In the 1.5 T sub-cohort, Bland–Altman analysis showed a marginal tendency in favor of automatic planning with slightly lower mean differences for LVESV, LVSV, and LVEF. The volumetric results derived from the automatically prescribed SAX image series presented a marginally increased scattering for the LVEDV in comparison to the manual planning. In contrast, the interval between the 95% limits-of-agreement (95%-LoA) of automated planning was found to be more constrained for the LVSV and the LVEF. Conversely, an opposing trend was observed for the RV volumetric results, showing consistently higher scatter width of 95%-LoA for automated planning, particularly for the RVEDV.

In the 3 T sub-cohort, a more pronounced trend favoring the automated prescription was observed compared to that in the 1.5 T sub-cohort. A comparable distribution of volumetric results was observed for the LVEDV, whereas all other volumetric values for the LV and RV exhibited a reduced distance of the 95%-LoA for the automated planning. Overall, the 3 T sub-cohort exhibited significantly lower mean deviations and reduced variability in volumetric parameters between scan 1 and scan 2 for both planning approaches.

## Discussion

This study investigated the reproducibility of RV and LV volumetric parameters derived from fully automatically prescribed image planes in direct comparison to manual planning. Subjective quality assessment of image planes showed high consistency for both planning approaches, with good to excellent quality across all plane orientations. The reproducibility of volumetric parameters was good to excellent. There was only a poor reproducibility for LVEF in the 1.5 T sub-cohort and a fair reproducibility in the 3 T sub-cohort for both planning methods. Overall, reproducibility was better in the 3 T sub-cohort compared to the 1.5 T sub-cohort, and ICC values were slightly higher compared to manual planning across both sub-cohorts. These trends were also observed in the Bland–Altman analysis.

The subjective assessment of plane quality showed statistically significant differences in quality ratings for automatically prescribed SAX image series in both sub-cohorts (1.5 T sub-cohort: *p* = 0.003; 3 T sub-cohort: *p* = 0.015). However, since the ratings ranged from 4.8/5 to 5/5, these marginal variances are most likely attributable to inter- and intra-reader variability rather than to evident changes in plane orientation quality. Further, high quality of automated planning has been validated in previous publications [22]. The high consistency and level of agreement between the automated and manual planning methods indicate high reproducibility and non-inferior performance of the automated method.

This trend was continued in the volumetric assessment of the RV and LV by good to excellent reproducibility of automated and manual prescription across all parameters except for the LVEF in both sub-cohorts and the RVEF in the 1.5 T sub-cohort. The impaired reproducibility of the LVEF was an unexpected finding, given that previous studies demonstrated a high reliability for all LV [21, 22] and RV [29] volumetric parameters. However, this phenomenon occurred in both planning approaches, which suggests a methodological cause for these observations. One aspect is that the EF is a calculated parameter depending on the EDV and ESV measurements. Errors and inaccuracies may be propagated and amplified due to the mathematical operations. This impact was already acknowledged in earlier investigations [21]. This study focused on the actual derived LV volumes (LVEDV and LVESV), which both demonstrated a good to high reproducibility. Further, large-scale multicenter studies on the LV parameters [7, 30] showed notable mean percentage deviations for the EF. These findings may have an increased impact on the investigated cohort of healthy volunteers because the variability of the EF is lower in comparison to patients with pathological EF. Consequently, this variation may have resulted in the observed impaired reproducibility. A second aspect relates to the time interval between subsequent follow-up scans. In the 1.5 T sub-cohort, where the second scan was conducted between two and five weeks after the first, LVEF showed only poor reproducibility, while RVEF demonstrated fair reproducibility. In contrast, the reproducibility of LVEF and RVEF was significantly superior in the 3 T sub-cohort, where the second scan was performed within 1–2 h after the first. Previous studies observed only minor bias for LVEF and RVEF, indicating a high degree of reproducibility [22, 29]. Nevertheless, in both studies, the second scan was performed immediately following the first with a short repositioning procedure. Physiological variation of cardiac volumes and function over an extended time period may have introduced variability into the reproducibility analysis of the 1.5 T sub-cohort. This is undermined by the fact that, in the 3 T sub-cohort, all cardiac functional parameters demonstrated higher ICC values, less inter-scan variation, and narrower 95%-LoA in the Bland-Altman analysis. The third aspect is that the volumetric analysis was performed fully automatically without expert correction. The reasons for this approach are discussed in the limitations section. This methodological difference may have caused deviating results compared to existing literature since previous studies utilized expert-corrected volumetric assessment [21, 22, 29]. In summary, ICC values of all volumetric parameters were comparable between automated and manual planning, with an emerging trend of increased reproducibility for the automated planning in the 3 T sub-cohort. This observation is undermined by the narrower 95%-LoA of LVSV and LVEF in both sub-cohorts and RVSV and RVEF in the 3 T sub-cohort derived from the automatically prescribed images. Despite the larger number of volunteers in the 1.5 T sub-cohort, this trend was not observed for the 1.5 T sub-cohort. It can be hypothesized that the influence of the physiological variability of cardiac parameters over the higher time interval between the exams in the 1.5 T sub-cohorts masks this trend, since the differences are only marginal.

This study has several limitations that must be mentioned. First, the study population included only healthy subjects with normal heart anatomy, and therefore, the applicability to patients with structural heart disease, such as congenital malformations, is limited. Second, the number of individuals in the study cohort was limited and recruited from a single center. However, the volunteer selection process incorporated an equal inclusion of both genders and pre-defined age groups to ensure a representative study population. A larger multi-center cohort would be beneficial to further validate the findings. Further, all CMR scans were performed on systems from a single vendor, and the tested automatic planning tool is a fully integrated vendor-specific software, which limits its generalizability. Further validation of our findings in a multi-center setting, including patient populations with cardiovascular heart disease, can enhance generalizability. Another aspect concerns the ICC analysis used to assess the test–retest reproducibility. It should be noted that in the 1.5 T subcohort, the results reflect intra-field strength reproducibility, whereas in the 3 T subcohort, it reflects inter-field strength reproducibility. Additional CMR scans at 3-T field strength would be well-suited to further assess the intra-field strength reproducibility at 3 T. Finally, the volumetric analysis was performed fully automatically without expert correction, which may have led to suboptimal volumetric results. However, the major advantage of this approach is that it eliminates the impact of any human intra-reader variability on the reproducibility analysis in this study [31], and automated segmentation and volumetric assessment are well validated with a broad clinical implementation [10, 32].

The clinical implementation of automated plane prescription for CMR has the potential to improve image acquisition and support technicians with a wide range of expertise. Additionally, automated planning may reduce the time required for plane prescription. However, this study did not investigate this potential because the proposed automated planning tool is currently in the prototype stage. The impact of automated planning on CMR scan times warrants further investigation in future releases. Automated plane prescription can increase standardization of CMR between different centers and enhance availability to smaller care units with fewer highly experienced personnel. This is important not only for daily clinical care but also for facilitating multicenter clinical trials that incorporate CMR. One example is the CONCERT-HF trial [33], which requires on-site and online training of technicians in order to ensure comparability of conducted CMR scans. Standardizing scanning protocols and image acquisition is a major challenge that often requires costly training and certification, which limits the number of centers that can participate in these valuable studies.

It can be concluded that automated plane prescription for CMR provides high-quality image planes, ensuring good to excellent reliability and reproducibility of volumetric parameters across both established field strengths.

## Supplementary information

ELECTRONIC SUPPLEMENTARY MATERIAL

## Abbreviations

2CH: 2-Chamber-view
3CH: 3-Chamber-view
4CH: 4-Chamber-view
95%-LoA: 95% Limits-of-agreement
CMR: Cardiac magnetic resonance imaging
EDV: End-diastolic volume
EF: Ejection fraction
ESV: End-systolic volume
LV: Left ventricle
MRI: Magnetic resonance imaging
RV: Right ventricle
SAX: Short-axis
SV: Stroke volume
