# Supplementary material for: Reproducibility of cardiac volumetric parameters derived from fully automatically prescribed image planes: a direct comparison to manual planning at 1.5-T and 3-T MRI
Source: Eur Radiol. 2026 Feb 21;36(7):5567–79. doi: 10.1007/s00330-026-12388-9 (PMC13282237; doi:10.1007/s00330-026-12388-9)
Supplement: Supplementary file 1 — ELECTRONIC SUPPLEMENTARY MATERIAL [file 330_2026_12388_MOESM1_ESM.pdf]

Reproducibility of cardiac volumetric parameters derived from fully automatically prescribed image planes: a direct comparison to manual planning at 1.5-T and 3-T MRI

ELECTRONIC SUPPLEMENTARY MATERIAL

Supplemental Table 1: Demographic data of the 1.5T and 3T sub-cohort

Demographic parameters

**1.5T sub-cohort**	**All participants**	**Men**	**Women**
Number	32	17	15
Age [years]	41 (23-68)	42 (23-68)	40 (23-64)
Weight [kg]	77.3 (± 11.1)	83.5 (± 8.0)	70 (± 9.8)
Height [m]	1.77 (± 0.09)	1.83 (± 0.07)	1.69 (± 0.05)
BMI [kg/m^2]	24.72 (± 2.72)	24.84 (± 2.02)	24.58 (± 3.42)
Heart Rate [bpm]	66.5 (± 12)	62 (± 11)	71 (± 12)
**3T sub-cohort**	**All participants**	**Men**	**Women**
Number	20	9	11
Age [years]	45.5 (24-71)	46 (24-71)	45 (24-64)
Weight [kg]	78.5 (± 14.8)	85.3 (± 13.2)	73 (± 13.9)
Height [m]	1.74 (± 0.1)	1.81 (± 0.08)	1.68 (± 0.08)
BMI [kg/m^2]	26.04 (± 4.57)	26.12 (± 4.02)	25.96 (± 5.19)
Heart Rate [bpm]	72 (± 9)	73 (± 9)	70 (± 9)

Supplemental Table 1: Demographic data of the 1.5T and 3T sub-cohort. Age values are presented as median with minimum and maximum values in brackets. Body weight, height, BMI and heart rate are presented as mean with standard deviation in brackets. BMI – body mass index.
